# Twenty-Five-Year Trends in Mortality Associated with *Clostridioides difficile* Infection Among Patients with Inflammatory Bowel Disease in the United States: A Population-Based Analysis of Demographic and Geographic Disparities

**DOI:** 10.3390/medsci14050511

**Published:** 2026-08-24

**Authors:** Ayesha Asghar, Abdullah Sultany, Shubhendu Bajpai, Amlish Gondal, Eshal Amir, Ayesha Kashaf, Sheeza Nawaz, Sahil Grover, Solomon Anighoro, Rahul Zain, Rewanth Katamreddy, Adam Breslin, Michelle Bernshteyn

**Affiliations:** 1 Department of Internal Medicine, Guthrie Robert Packer Hospital, 1 Guthrie Sq, Sayre, PA 18840, USA; ayesha.asghar@guthrie.org (A.A.); shubhendu.bajpai@guthrie.org (S.B.); sheeza.nawaz@guthrie.org (S.N.); sahil.grover@guthrie.org (S.G.); solomon.anighoro@guthrie.org (S.A.); 2 Department of Gastroenterology and Hepatology, Guthrie Robert Packer Hospital, Sayre, PA 18840, USA; 3 FMH College of Medicine and Dentistry, Lahore 54000, Pakistan; eshalamir05@gmail.com; 4 Gujranwala Medical College, Gujranwala 52250, Pakistan; 5 Department of Pulmonary and Critical care Medicine, Guthrie Robert Packer Hospital, Sayre, PA 18840, USA

**Keywords:** inflammatory bowel disease, *Clostridioides difficile*, mortality, CDC WONDER, joinpoint regression, health disparities, COVID-19

## Abstract

Background: Individuals with inflammatory bowel disease (IBD) are at substantially increased risk for *Clostridioides difficile* infection (CDI), which leads to significantly higher morbidity and mortality compared to the general population. However, comprehensive national-level analyses of long-term mortality trends in this population remain limited. This study examines mortality trends associated with IBD and CDI in the United States from 1999 to 2023. Methods: This descriptive study utilized the CDC WONDER Multiple Cause-of-Death database. Deaths involving IBD (ICD-10: K50, K51) and CDI (A04.7) were identified among adults aged 25 years and older. Age-adjusted mortality rates (AAMRs) per 100,000 population were calculated with 95% confidence intervals and stratified by sex, race/ethnicity, urbanization, and census region. Joinpoint regression was applied to estimate the annual percent change (APC) in mortality. Results: Between 1999 and 2023, 76,084 deaths were recorded. Medical facilities accounted for 46% of deaths, followed by decedents’ homes (28.3%) and nursing home/long-term care facilities (16.5%). Overall mortality declined gradually from 1999 to 2018 (APC: −0.23, *p* < 0.05), increased sharply through 2021 (APC: +12.75, *p* < 0.05), and was then followed by a non-significant change through 2023 (APC: −2.69; 95% CI: −8.24 to 3.19), consistent with a plateau. Men consistently exhibited higher AAMRs than women. Non-Hispanic White individuals had the highest AAMRs (1.844 in 2023), while Non-Hispanic Black individuals experienced a sustained increase from 2016 onward (APC: +7.11, *p* < 0.05). Hispanic mortality increased steadily throughout the study period (APC: +1.31, *p* < 0.05). Rural populations had higher overall AAMRs than urban populations. The Midwest recorded the highest regional AAMRs by 2023 (1.867). Conclusions: Mortality increased significantly between 2018 and 2021, coinciding with the COVID-19 pandemic, though our study design cannot prove causation. Disparities by race/ethnicity, urbanization, and region persisted. These findings underscore the need for ongoing antibiotic stewardship, equitable healthcare access, and targeted public health interventions for this vulnerable population.

## 1. Introduction

*Clostridioides difficile* infection (CDI) continues to be a major healthcare-associated infection in the United States, with an estimated 453,000 cases and 29,300 deaths associated with CDI annually [1]. There has been significant epidemiological shift over the past two and a half decades for CDI. With the emergence and rapid dissemination of the hypervirulent NAP1/BI/027 strain, this has led to a marked increase in disease severity, refractory cases, and overall mortality in early 2000s and thus, has consequently led to an increased national burden of CDI, reaching its highest incidence between the late 2000s and early 2010s. Over the ensuing decade, better public health and clinical interventions have led to the decline in hospital-related CDI after stewardship and infection-control efforts. However, this decrease in hospital-related cases has been replaced by the shift toward community-acquired CDI in recent years, with the increasing effect on previously low-risk populations [1,2].

Patients with inflammatory bowel disease (IBD) carry a substantially elevated risk of CDI, with population-based data demonstrating a nearly five-fold increase in CDI incidence compared to the general population [2,3]. This heightened susceptibility is driven by a confluence of factors, including chronic intestinal inflammation, gut microbiome dysbiosis, frequent antibiotic exposure, and the widespread use of immunosuppressive therapies, such as corticosteroids and biologic agents [1,2].

The clinical consequences of concurrent CDI in IBD are severe. Hospitalized IBD patients with CDI face approximately four-fold higher mortality compared to those admitted for IBD alone [2]. CDI in this population is also associated with increased rates of colectomy, prolonged hospital stays, higher healthcare costs, and a greater likelihood of recurrent infection [3,4]. The published literature has demonstrated that ulcerative colitis confers a disproportionately elevated risk of CDI-related complications and mortality compared to Crohn’s disease [2,5].

Over the past two decades, the epidemiological landscape of CDI has evolved considerably. National efforts in antibiotic stewardship and infection control contributed to a decline in healthcare-associated CDI between 2011 and 2017 [1]. However, the emergence of the COVID-19 pandemic in 2020 introduced unprecedented disruptions to healthcare delivery, infection prevention practices, and patient care pathways, with complex and potentially divergent effects on CDI epidemiology [6,7]. While some studies reported decreased CDI incidence during the pandemic period due to enhanced hygiene measures and reduced elective procedures, others documented worsened CDI outcomes, including higher inpatient mortality among those who did develop the infection [8,9]. In this analysis, we used death-certificate data that count all deaths due to this increasing burden, including deaths at home, in hospice, and in nursing homes, specifically for people who had both IBD and CDI, over 25 years [1,5].

Despite the recognized burden of CDI in IBD, comprehensive national-level analyses of long-term mortality trends in this specific population remain limited. To our knowledge, no prior study has examined national mortality trends specifically in patients who have both IBD and CDI over a 25-year period across demographic and geographic groups [5]. Understanding how mortality has evolved over time, as well as how it varies across demographic subgroups, geographic regions, and urbanization levels, is essential for informing targeted public health interventions. This study aimed to examine the mortality trends associated with IBD and CDI in the United States from 1999 to 2023 using the CDC WONDER database, with stratification by sex, race/ethnicity, urbanization status, and census region.

## 2. Methods

### 2.1. Study Setting and Population

This descriptive study utilized data from the CDC WONDER (Centers for Disease Control and Prevention Wide-Ranging Online Data for Epidemiologic Research) database, examining deaths from 1999 to 2023 attributed to inflammatory bowel disease with *Clostridioides difficile* colitis. Cases were identified using International Statistical Classification of Diseases and Related Health Problems, 10th Revision (ICD-10) codes: K50 for Crohn’s disease, K51 for ulcerative colitis, and A04.7 for enterocolitis due to *Clostridioides difficile* [10,11].

The dataset comprises cause-of-death information from death certificates for inflammatory bowel disease across all fifty states and has been previously used to examine mortality trends in this condition. The Multiple Cause-of-Death public-use data enabled identification of deaths where inflammatory bowel disease (either Crohn’s disease or ulcerative colitis) was listed as an underlying cause, and *Clostridioides difficile* colitis was identified as a contributing factor.

Data were restricted to adults aged 25 years and older to minimize misclassification of pediatric and young adult deaths, which may have distinct etiologies and risk profiles. Institutional review board approval was not required, as the study used publicly available, de-identified government data and adhered to the STROBE (Strengthening the Reporting of Observational Studies in Epidemiology) guidelines [12].

### 2.2. Data Extraction

Abstracted data included the population size, year, location of death, demographic variables, urban–rural classification, region, and state. Demographic variables comprised sex, age, and race/ethnicity. Death locations included medical facilities (outpatient, inpatient, emergency room, death on arrival, or unknown), home, hospice, and nursing home or long-term care facilities. Race/ethnicity was primarily categorized as Hispanic/Latino, Non-Hispanic Black/African American, and Non-Hispanic White. These classifications were based on information reported on death certificates, consistent with prior analyses using the CDC WONDER database.

The population was categorized as urban (large metropolitan areas with populations of one million or more), medium or small metropolitan areas (populations between 50,000 and 999,999), and rural (populations less than 50,000) according to the 2013 United States Census classification and the National Center for Health Statistics Urban-Rural Classification Scheme [13]. Urbanization analyses were restricted to 1999–2020 because the NCHS urban–rural classification and population estimates were revised after 2020, which prevents direct comparison with earlier years [13]. U.S. regions were classified as northeast, Midwest, south, or west, following U.S. Census Bureau guidelines.

### 2.3. Statistical Analysis

In order to examine national trends in inflammatory bowel disease and *Clostridioides difficile* colitis-related mortality, crude and age-adjusted mortality rates (AAMRs) per 100,000 population from 1999 to 2023 by year, sex, race/ethnicity, state, and urban/rural status were calculated with 95% confidence intervals (CIs). AAMRs were standardized to the US population in the year 2000 [14]. Records of incomplete or missing demographic or cause-of-death data were excluded from subgroup analyses for maintenance of data integrity.

For the quantification of national annual trends over the time period of 1999–2023 in IBD and *C. difficile* colitis-related mortality, the Joinpoint Regression Program (Version 5.4.0) [15] was used for the determination of annual percent change (APC) with 95% CIs. The log-linear regression models were applied, where the temporal trends showed significant variations in AAMRs over time. Two-tailed *t*-tests were applied, and APCs were considered based on the slope describing the change in mortality, where a *p*-value of < 0.05 was considered statistically significant.

## 3. Results

**Figure 1:**
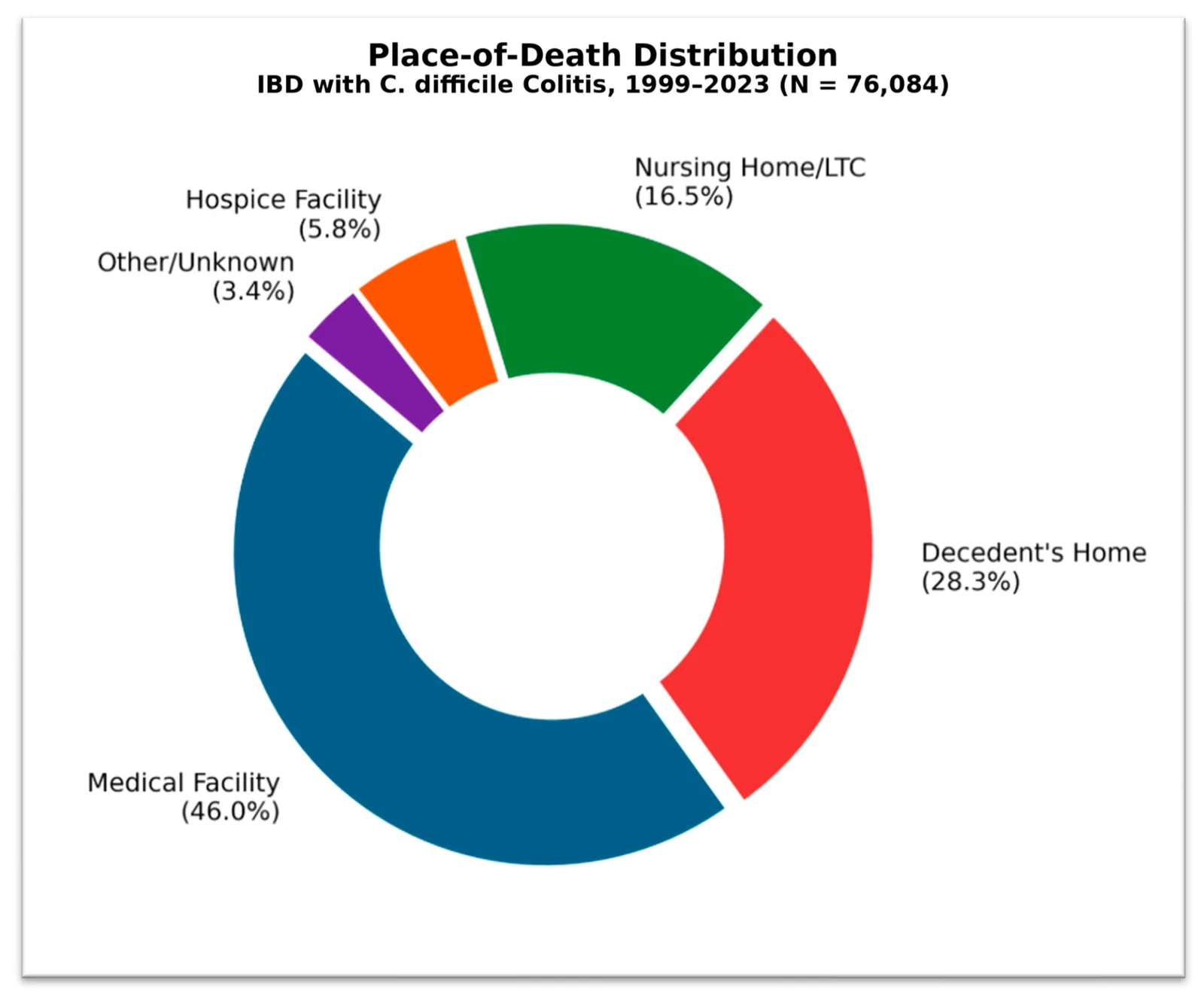
Place-of-death distribution for IBD with *C. difficile* colitis in the United States, 1999–2023 (N = 76,084).

A total of 76,084 deaths occurred due to inflammatory bowel disease with *Clostridioides difficile* colitis during the time span of 1999 to 2023. According to the place-of-death data, medical facilities had the highest mortality (46%) overall, followed by decedent’s homes (28.3%), nursing home/long-term care facilities (16.5%), and hospice facilities (5.8%), with the rest being unknown (Figure 1).

*Overall Mortality Trends*

**Figure 2:**
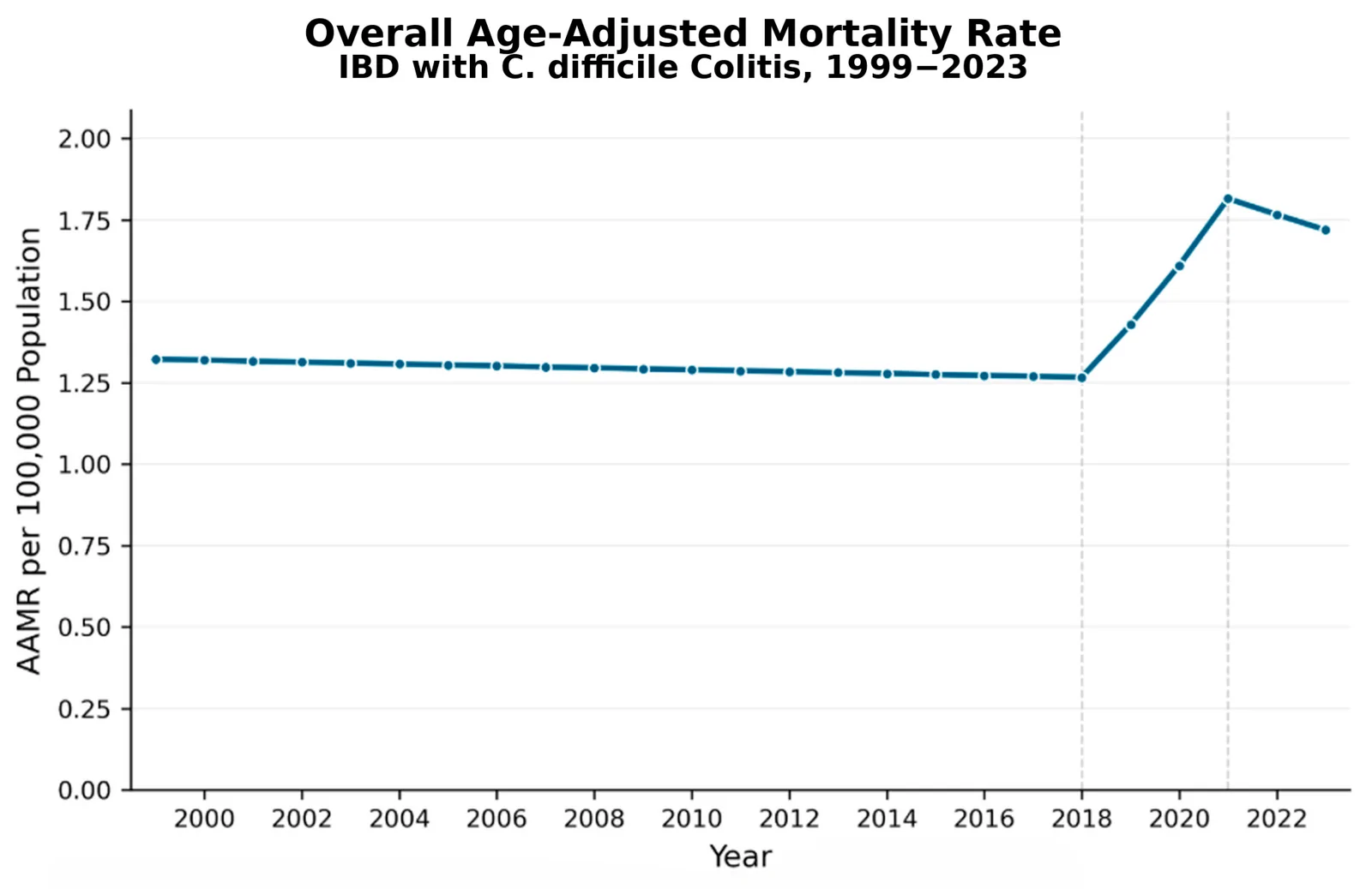
Overall age-adjusted mortality rate (AAMR) per 100,000 population for IBD with *C. difficile* colitis, 1999–2023. Joinpoints identified at 2018 and 2021.

During the time period of 1999 to 2023, a total of 76,084 deaths occurred due to inflammatory bowel disease with *Clostridioides difficile* colitis. In 1999, the AAMR was 1.322 (95% CI: 1.269 to 1.375), and by 2023 it had risen to 1.685 (95% CI: 1.634 to 1.735). Regarding the annual trends, the mortality initially decreased slightly from 1999 to 2018 (APC: −0.226; 95% CI: −0.4478 to −0.003), followed by a sharp rise till 2021 (APC: 12.747; 95% CI: 5.847 to 20.096), which then plateaued through 2023 (APC: −2.691; 95% CI: −8.235 to 3.189; non-significant) (Figure 2).

*Mortality Trends Regarding Gender*

**Figure 3:**
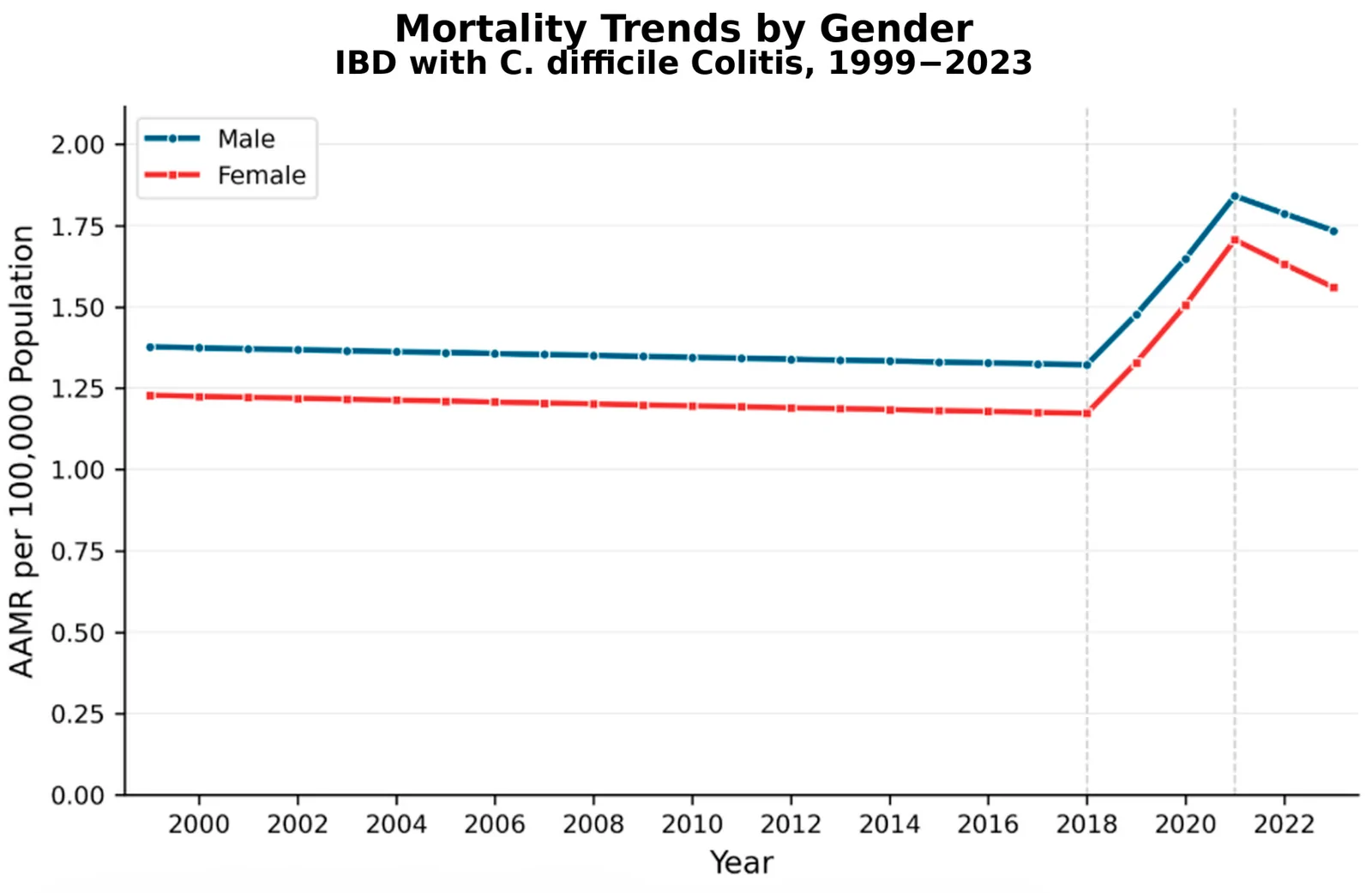
AAMRs per 100,000 population by gender for IBD with *C. difficile* colitis, 1999–2023.

On the basis of gender stratification, men had consistently higher AAMRs compared to women throughout the study period. In 2023, the AAMR for men was 1.736 (95% CI: 1.661 to 1.812), while for women, it was 1.559 (95% CI: 1.495 to 1.623). There was an initial decline in the mortality till 2018 for both genders. Mortality then increased till 2021, with APCs of 11.670 (95% CI: 4.064 to 19.832) and 13.311 (95% CI: 4.721 to 22.605) for men and women, respectively. It was again then followed by a decrease till 2023, with men having an APC of −2.942 (95% CI: 8.971 to 3.486), while for women, their APC was −4.374 (95% CI: −11.070 to 2.826) (Figure 3).

*Mortality Trends Regarding Race/Ethnicity*

**Figure 4:**
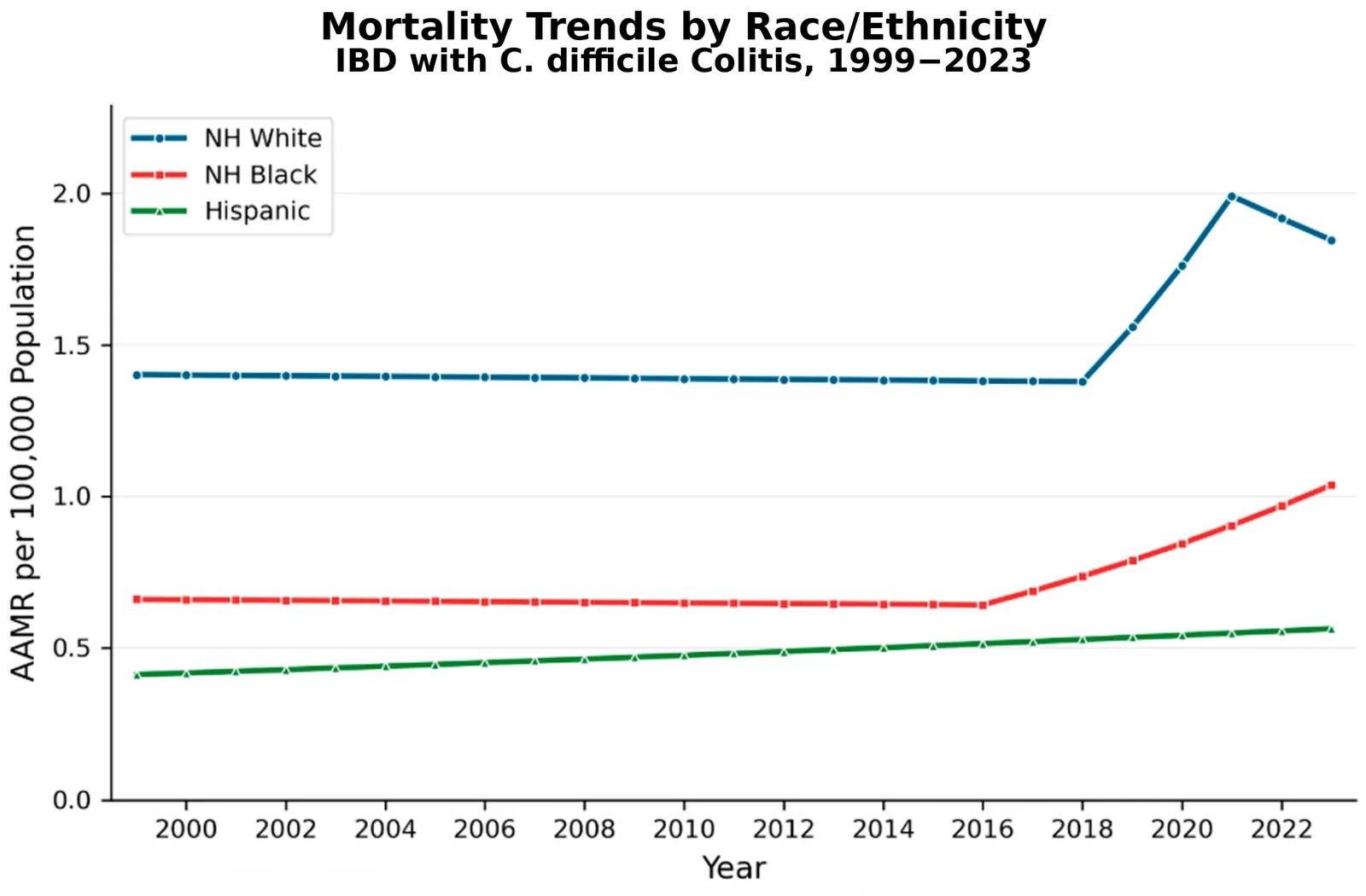
AAMRs per 100,000 population by race/ethnicity for IBD with *C. difficile* colitis, 1999–2023.

On the basis of stratification by race, the NH White population had the highest mortality throughout the study period, followed by NH Black and African Americans and Hispanics with the AAMRs in 2023 of 1.844, 1.038, and 0.563, respectively. For NH White, there was an initial decrease from 1999 to 2018 followed by a sharp rise till 2021 (APC: 13.042; 95% CI: 4.504 to 22.277), which then again decreased till 2023 (APC: −3.728; 95% CI: −10.386 to 3.425). For NH Black or African Americans, the AAMRs slightly declined from 1999 to 2016 (APC: −0.166; 95% CI: −1.183 to 0.862), followed by a sharp rise till 2023 (APC: 7.107; 95% CI: 3.884 to 10.429). For Hispanics, the mortality increased consistently throughout the study period (APC: 1.314; 95% CI: 0.374 to 2.264) (Figure 4).

*Mortality Trends Regarding Urbanization*

**Figure 5:**
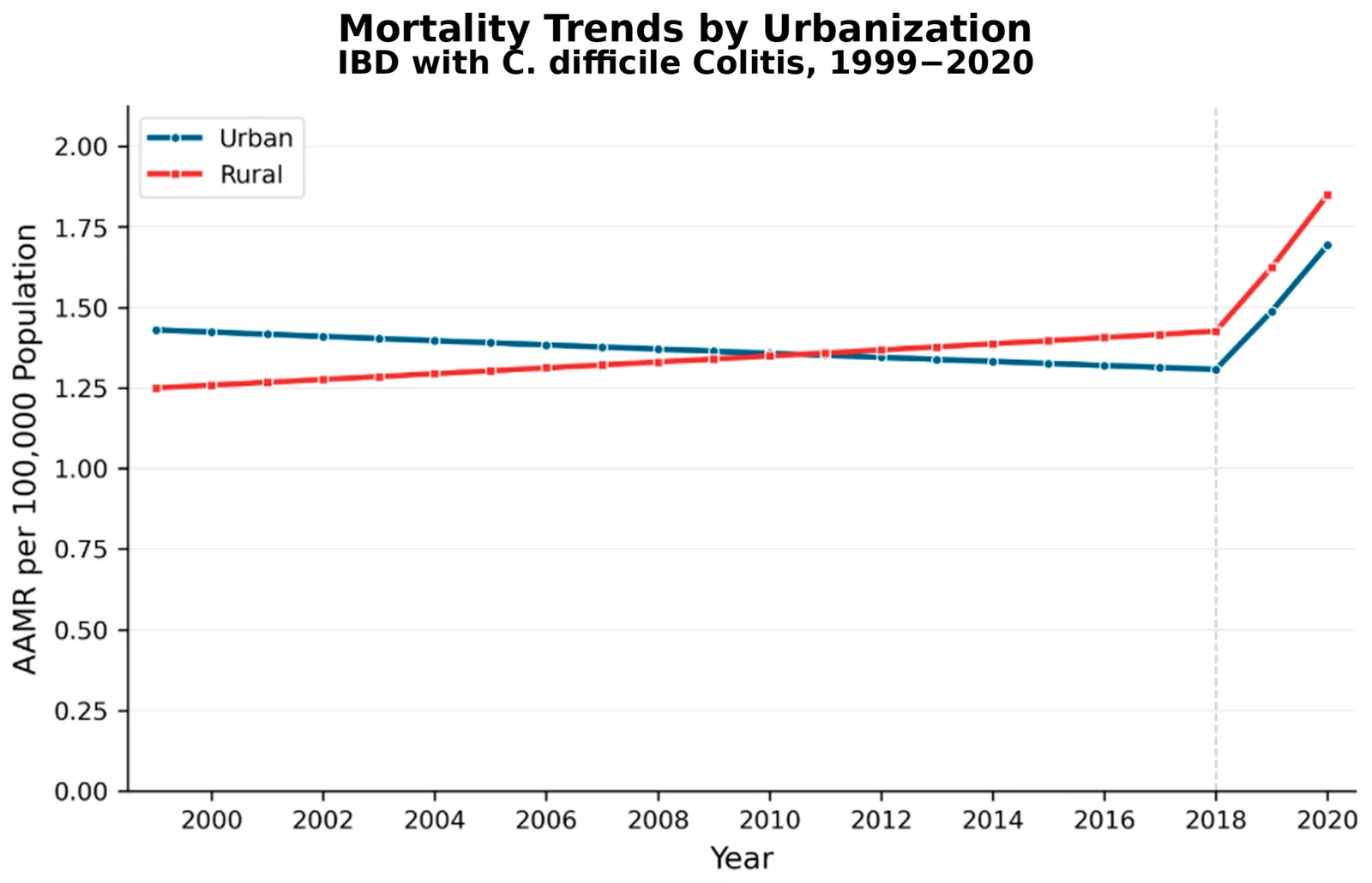
AAMRs per 100,000 population by urbanization for IBD with *C. difficile* colitis, 1999–2020.

On the basis of urbanization, over the period from 1999 to 2020, the overall AAMRs were higher for the rural population (1.390) compared to the urban population (1.303). For rural areas, the AAMR showed a steady increase from 1999 to 2018 (APC: 0.695; 95% CI: 0.364 to 1.028), followed by a spike till 2020 (APC: 13.863; 95% CI: 3.519 to 25.239). For urban areas, there was a slight decrease in the mortality from 1999 to 2018 (APC: −0.471; 95% CI: −0.747 to −0.194), followed by a sharp increase till 2020 (APC: 13.795; 95% CI: 5.116 to 23.191) (Figure 5).

*Mortality Trends Regarding Census Region*

**Figure 6:**
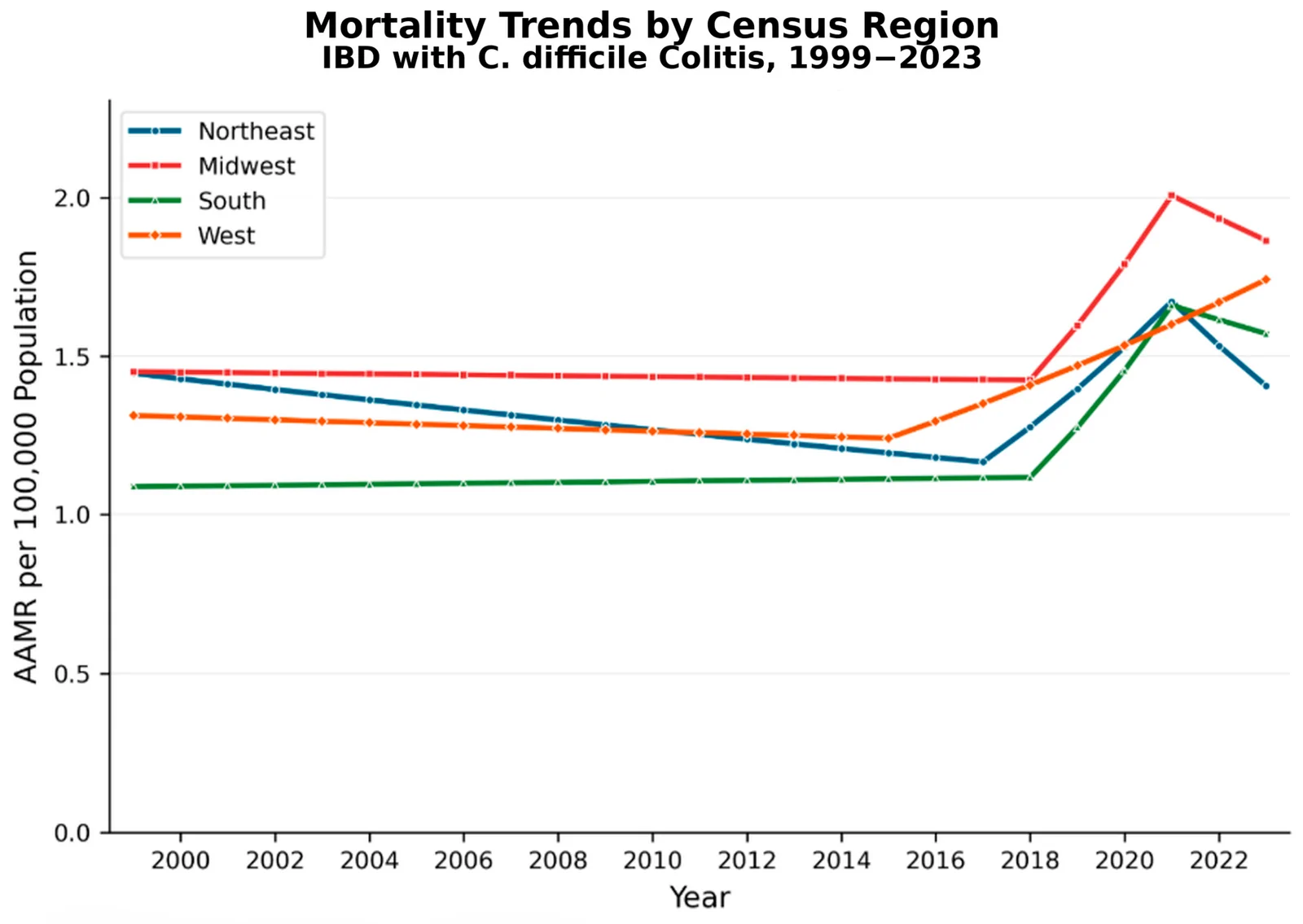
AAMRs per 100,000 population by U.S. census region for IBD with *C. difficile* colitis, 1999–2023.

On the basis of regional stratification, the highest AAMR in 2023 was in the Midwest (1.867), followed by the west (1.743), the south (1.572), and the northeast (1.406). For the Midwest, the mortality decreased initially from 1999 to 2018 (APC: −0.097; 95% CI: −0.551 to 0.359), which then sharply increased till 2021 (APC: 12.115; 95% CI: −2.013 to 28.280), followed by a decline till 2023 (APC: −3.605; 95% CI: −14.709 to 8.945). For the west, the AAMRs initially declined from 1999 to 2015 (APC: −0.351; 95% CI: −1.083 to 0.386), followed by a steady increase till 2023 (APC: 4.329; 95% CI: 2.606 to 6.081). For the south, there was a steady mortality trend from 1999 to 2018, followed by a spike till 2021 (APC: 14.110; 95% CI: 4.606 to 24.477), which then decreased till 2023 (APC: −2.729; 95% CI: −9.934 to 5.051). For the northeast, after a consistent decline from 1999 to 2017, the mortality increased till 2021 (APC: 9.433; 95% CI: 3.991 to 15.159), followed by a decrease again till 2023 (APC: −8.326; 95% CI: −16.776 to 0.982) (Figure 6).

## 4. Discussion

This study provides a comprehensive analysis of mortality trends in patients with IBD and concurrent CDI across the United States over a 25-year period. The findings reveal a complex temporal pattern characterized by an initial gradual decline in mortality, followed by a dramatic surge around 2018 to 2021, and a subsequent partial decline through 2023. These trends were consistent across nearly all demographic and geographic subgroups, though notable disparities emerged by race/ethnicity, urbanization, and region.

The overall age-adjusted mortality rate increased from the start to the end of the study period despite an initial period of gradual decline spanning nearly two decades. This early downward trend likely reflects the cumulative benefits of improved CDI management strategies, including the adoption of oral vancomycin and fidaxomicin as first-line therapies, advances in antibiotic stewardship programs, and enhanced infection prevention measures in healthcare facilities [16,17,18]. The national burden of CDI decreased by approximately 24% between 2011 and 2017, driven largely by reductions in healthcare-associated infections [1]. These gains appear to have been mirrored in the IBD population during the same period.

The sharp increase in mortality observed from approximately 2018 to 2021 represents the most striking finding of this analysis. The annual percent change during this interval exceeded 12% for the overall population, indicating a rapid and clinically significant reversal of prior gains. This increase coincided with the COVID-19 pandemic, which may have contributed. However, because this is a death-certificate analysis, we cannot prove causation. National surveillance data even showed no significant change in CDI trends right after March 2020, and the pandemic’s effect on CDI appears mixed—lower infection rates in some settings but higher inpatient mortality. Although the pandemic led to enhanced infection control measures that may have reduced overall CDI incidence in some settings, the impact on CDI outcomes was markedly different [8]. National data demonstrated that CDI patients experienced significantly higher inpatient mortality during the pandemic period compared to the pre-pandemic era [9]. Several mechanisms likely contributed to this paradox but as this is an ecological analysis, we cannot establish that COVID-19 or any single factor caused these changes.

First, the pandemic caused widespread disruption to routine healthcare delivery for patients with chronic diseases, including IBD [2]. Delays in outpatient visits, deferred endoscopic evaluations, and interruptions in maintenance therapy may have led to disease flares and increased vulnerability to CDI [2]. Second, the extensive use of broad-spectrum antibiotics for suspected or confirmed COVID-19, particularly during the early pandemic period, may have promoted gut dysbiosis and facilitated *C. difficile* colonization and infection [3,6]. Third, COVID-19 itself has been associated with intestinal inflammation and microbiome disruption through direct viral invasion of the gastrointestinal tract via the angiotensin-converting enzyme 2 receptor [3]. Population-level data from Wales showed that CDI cases were twice as likely to have had prior COVID-19 infection as controls [19]. Fourth, diverting healthcare resources to pandemic response may have weakened antibiotic stewardship programs and infection prevention infrastructure, particularly in overburdened facilities [18].

The observation that mortality appeared to plateau after 2021 without a statistically significant decline and remained above pre-pandemic levels, though not returning to pre-pandemic levels, suggests a partial recovery as healthcare systems stabilized and pandemic-related disruptions attenuated. However, the persistence of elevated mortality rates through 2023 indicates that the pandemic’s impact on this vulnerable population has not been fully reversed.

The finding that men had consistently higher mortality rates than women throughout the study period aligns with broader epidemiological patterns in CDI. Male sex has been identified as a risk factor for adverse outcomes in both CDI and IBD independently [2]. The parallel temporal trends observed in both sexes, with similar timings of the pandemic-era surges and subsequent decline, suggest that the underlying drivers of mortality changes affected both groups comparably.

Racial and ethnic disparities in mortality represent a particularly concerning finding. The Non-Hispanic White population had the highest absolute mortality rates throughout the study period, consistent with the higher prevalence of IBD in this group [20]. However, the trajectory among Non-Hispanic Black or African American individuals is notable for a sustained and accelerating increase in mortality from 2016 through 2023, without the post-2021 decline observed in other groups. This persistent upward trend may reflect compounding effects of structural inequities in healthcare access, higher rates of CDI among hospitalized African American IBD patients, and social determinants of health that contribute to microbiome dysbiosis and adverse infection outcomes [21,22]. The steady increase in mortality among Hispanic individuals throughout the entire study period similarly warrants attention, potentially reflecting the rising incidence of IBD in this population combined with barriers to specialty care [20,23].

The urban–rural analysis revealed that rural populations had higher overall mortality rates than urban populations. This disparity is consistent with documented differences in healthcare access for IBD patients in rural areas, including lower rates of specialist gastroenterology visits, reduced access to multidisciplinary care, and higher rates of emergency department utilization and hospitalization [24,25]. Rural IBD patients are less likely to receive advanced therapies and more likely to present with complications requiring surgical intervention [25,26]. The pronounced mortality spike observed in both rural and urban areas around 2020 suggests that the pandemic’s impact was geographically widespread, though the pre-existing vulnerability of rural populations may have amplified its effects.

Regional variation in mortality trends provides additional insight into the heterogeneity of this public health challenge. The Midwest region had the highest mortality rates by the end of the study period, followed by the west, south, and northeast. The Midwest’s elevated burden may be related to its lower density of gastroenterologists among IBD patients and the higher proportion of rural communities with limited access to specialty care [27]. The western region’s steady increase in mortality from 2015 onward, without the post-2021 decline seen in other regions, is particularly concerning and may reflect unique demographic shifts, including growing IBD prevalence in previously lower-incidence populations. The northeast, despite having the lowest mortality rates by 2023, experienced a significant pandemic-era surge followed by the steepest decline, possibly reflecting a greater concentration of academic medical centers and a more rapid recovery of healthcare infrastructure.

The predominance of medical facility deaths, accounting for nearly half of all mortality, underscores the severity of CDI in hospitalized IBD patients. This finding is consistent with data showing that the combination of IBD and CDI during hospitalization carries a mortality risk approximately four-fold higher than IBD alone and two-fold higher than CDI alone [1]. The substantial proportion of deaths occurring at home and in nursing facilities highlights the need for improved outpatient and long-term care management strategies for this population.

Several actionable implications emerge from these findings. First, the pandemic-era mortality surge underscores the critical importance of maintaining robust antibiotic stewardship programs even during public health emergencies. Meta-analyses have demonstrated that antimicrobial stewardship programs reduce CDI incidence by 30% to 50%, and any erosion of these programs during crises may have outsized consequences for vulnerable populations [17]. Second, ensuring continuity of IBD care during healthcare disruptions, including through telemedicine and remote monitoring, is essential to prevent disease flares that predispose to CDI [2]. Third, the persistent racial and ethnic disparities demand targeted interventions to improve healthcare access and CDI prevention in minority populations [22]. Fourth, the rural-urban gap underscores the need for innovative care-delivery models, such as hub-and-spoke specialist consultation networks, to extend IBD expertise to underserved areas [25,26]. Fifth, the rising prevalence of IBD in the United States, now estimated to affect over 2.39 million Americans, means that the population at risk for CDI-related mortality will continue to grow, necessitating sustained investment in prevention and treatment infrastructure [20].

This study has several limitations inherent to the use of the CDC WONDER database. First, mortality data are derived from death certificates, which depend on the accuracy of cause-of-death coding by certifying physicians. Death-certificate data depend on how doctors code cause of death. We cannot tell if CDI was the actual cause of death or just present at the same time. Coding and testing practices changed over 25 years—including the 2017 A04.7 code split and the switch between the two CDC WONDER files—which can affect the trend [2,28,29]. Misclassification of either IBD or CDI as a contributing cause of death may lead to underestimation or overestimation of the true burden. Second, the database has no clinical details (disease severity, medications like steroids/biologics, laboratory values other illnesses, hospital details, or income/social factors), limiting the ability to adjust for confounders or identify specific risk factors driving mortality trends. Third, as this is an ecological study, we cannot prove COVID caused the changes. Fourth, the urbanization data were available only through 2020, preventing assessment of rural-urban trends during the later pandemic and post-pandemic periods. Fifth, the analysis was not run on the basis of IBD subtypes for comparison, so the mortality difference between UC and CD (i.e., either of which has higher mortality) cannot be predicted. Sixth, race and ethnicity data on death certificates are reported by informants or funeral directors, which may introduce misclassification, particularly for Hispanic and multiracial individuals. Despite these limitations, the CDC WONDER database provides a nationally representative, population-based dataset that enables the identification of broad mortality trends and disparities over extended time periods.

## 5. Conclusions

This 25-year analysis of national mortality data reveals that deaths associated with IBD and CDI in the United States followed a pattern of gradual decline from 1999 to 2018, followed by a sharp increase through 2021 that subsequently plateaued at elevated levels. The COVID-19 pandemic may have contributed to this surge, although other factors cannot be excluded, like disruptions to healthcare delivery, increased antibiotic use, and direct effects on gut health. Persistent disparities by race/ethnicity, urbanization, and geographic region were observed and warrant further investigation into their causes. Although our data cannot establish causation, these patterns suggest that equitable healthcare access and antibiotic stewardship may be useful targets for future study and intervention, that are essential steps to reverse these concerning trends and reduce the burden of CDI-related mortality in the IBD population.

## Data Availability

Data are publicly available through CDC WONDER (https://wonder.cdc.gov), accessed on 21 January 2026.
